# Activation of Transducin by Bistable Pigment Parapinopsin in the Pineal Organ of Lower Vertebrates

**DOI:** 10.1371/journal.pone.0141280

**Published:** 2015-10-22

**Authors:** Emi Kawano-Yamashita, Mitsumasa Koyanagi, Seiji Wada, Hisao Tsukamoto, Takashi Nagata, Akihisa Terakita

**Affiliations:** 1 Department of Biology and Geosciences, Graduate School of Science, Osaka City University, Sugimoto, Sumiyoshi-ku, Osaka, 558–8585, Japan; 2 The OCU Advanced Research Institute for Natural Science and Technology (OCARINA), Osaka City University, Sugimoto, Sumiyoshi-ku, Osaka, 558–8585, Japan; 3 Japan Science and Technology Agency (JST), Precursory Research for Embryonic Science and Technology (PRESTO), Tokyo, Japan; Universitat Politècnica de Catalunya, SPAIN

## Abstract

Pineal organs of lower vertebrates contain several kinds of photosensitive molecules, opsins that are suggested to be involved in different light-regulated physiological functions. We previously reported that parapinopsin is an ultraviolet (UV)-sensitive opsin that underlies hyperpolarization of the pineal photoreceptor cells of lower vertebrates to achieve pineal wavelength discrimination. Although, parapinopsin is phylogenetically close to vertebrate visual opsins, it exhibits a property similar to invertebrate visual opsins and melanopsin: the photoproduct of parapinopsin is stable and reverts to the original dark states, demonstrating the nature of bistable pigments. Therefore, it is of evolutionary interest to identify a phototransduction cascade driven by parapinopsin and to compare it with that in vertebrate visual cells. Here, we showed that parapinopsin is coupled to vertebrate visual G protein transducin in the pufferfish, zebrafish, and lamprey pineal organs. Biochemical analyses demonstrated that parapinopsins activated transducin *in vitro* in a light-dependent manner, similar to vertebrate visual opsins. Interestingly, transducin activation by parapinopsin was provoked and terminated by UV- and subsequent orange-lights irradiations, respectively, due to the bistable nature of parapinopsin, which could contribute to a wavelength-dependent control of a second messenger level in the cell as a unique optogenetic tool. Immunohistochemical examination revealed that parapinopsin was colocalized with Gt2 in the teleost, which possesses rod and cone types of transducin, Gt1, and Gt2. On the other hand, in the lamprey, which does not possess the Gt2 gene, *in situ* hybridization suggested that parapinopsin-expressing photoreceptor cells contained Gt1 type transducin GtS, indicating that lamprey parapinopsin may use GtS in place of Gt2. Because it is widely accepted that vertebrate visual opsins having a bleaching nature have evolved from non-bleaching opsins similar to parapinopsin, these results implied that ancestral bistable opsins might acquire coupling to the transducin-mediated cascade and achieve light-dependent hyperpolarizing response of the photoreceptor cells.

## Introduction

In non-mammalian vertebrates, the pineal organs contain photoreceptor cells and receive light utilized for non-visual functions. The pineal organs of lampreys and teleosts detect the ratio of ultraviolet (UV) light to visible light; that is, they possess the ability of wavelength discrimination, similar to the pineal related organs, the frog frontal organ and lizard parietal eye [1–5]. We found that parapinopsin, which was originally identified in the catfish pineal and parapineal organs [6], is a UV-sensitive pigment underlying the wavelength discrimination in the lamprey pineal organ [7]. In addition, we identified the parapinopsin gene expression in the pineal and related organs of various non-mammalian vertebrates [7–9].

Parapinopsin is similar in amino acid sequence to and phylogenetically close to vertebrate visual opsins. However, our spectroscopic analysis showed that parapinopsin has a molecular property different from that of vertebrate visual opsins and similar to that of invertebrate visual opsins [7]. In general, opsins are converted to photoproducts in a light-dependent manner, which activate G protein [10]. The photoproducts of vertebrate visual opsins are unstable, release their chromophores, and consequently bleach. However, the photoproduct of parapinopsin is stable, does not release its chromophore and reverts to the original dark state by subsequent light-absorption, similar to invertebrate visual opsins and melanopsin, showing a bistable nature [11–14]. Parapinopsin-expressing photoreceptor cells in the lamprey pineal organ hyperpolarize to light [7], similar to vertebrate visual cells containing visual pigments. The type of molecules that interact with parapinopsin, which has intermediate features of vertebrate and invertebrate visual pigments [13] to transduce light information, requires investigation.

We recently revealed that the lamprey parapinopsin binds to β-arrestin in a light-dependent manner, in contrast to the vertebrate visual pigments, which bind to visual arrestins [15]. The β-arrestin is known to bind to G protein-coupled receptors other than opsin-based pigments [16], indicating that the arrestin-related shut-off mechanism for parapinopsin is different from that of vertebrate visual opsins involving visual arrestins. However, interestingly, we immunohistochemically found that parapinopsin was colocalized with transducin in the lamprey pineal photoreceptor cells [15], similar to vertebrate visual cells, rods and cones, suggesting that the bistable pigment parapinopsin might activate the transducin-mediated phototransduction cascade. We previously reported that parapinopsin activated Gi-type G protein in a light-dependent manner [13,17]; however, it is unclear whether parapinopsin actually activates transducin, which is classified into Gi-type G protein, *in vitro*.

Here we investigated G proteins coupling to parapinopsin, both biochemically and immunohistochemically, in the teleost and lamprey pineal organs. We analyzed whether parapinopsin activated transducin *in vitro* and further investigated the effect of the bistable nature of parapinopsin on G protein activation. Most vertebrates possess two kinds of transducins, Gt1 and Gt2, which are distributed in rods and cones, respectively, whereas the lamprey possesses a unique transducin GtL, which is not clearly classified into Gt1 or Gt2 groups, in addition to Gt1 type transducin, GtS [18]. Therefore, we immunohistochemically identified the kind of transducin coupled with parapinopsin in teleost and lamprey pineal organs.

## Materials and Methods

### Animals

Pufferfish, *Takifugu rubripes* and river lampreys, *Lethenteron camtschaticum* were commercially obtained. Zebrafish, *Danio rerio*, were obtained from the Zebrafish International Resource Center (ZIRC).

### Ethics Statement

This experiment was approved by the Osaka City University animal experiment committee (#S0032) and complied with the Regulations on Animal Experiments from Osaka City University.

### Expression and Purification of Parapinopsin

The pigment expression in HEK293S cells and pigment purification was performed as described previously [7]. Briefly, the cDNAs of pufferfish parapinopsin 1 (PP1, AB626964 (accession number in the DDBJ) [8]), zebrafish parapinopsin1 (PP1, AB626966 [8]), and lamprey parapinopsin (AB116380 [7]) were tagged with the epitope sequence for the monoclonal antibody rho 1D4 (ETSQVAPA). The tagged cDNA was inserted into the plasmid vector pcDNA3.1 (Invitrogen). To reconstitute the pigment, the expressed proteins were incubated with excess 11-*cis* retinal overnight. The pigments were then extracted with a detergent, 1% dodecyl β-D-maltoside, in 50 mM HEPES buffer (pH 6.5) containing 140 mM NaCl (buffer A). For purification, the pigments in the crude extract were bound to 1D4-agarose, washed with 0.02% dodecyl β-D-maltoside in buffer A (buffer B), and eluted with buffer B that contained the 1D4 peptide. For the control experiment, the bovine rhodopsin was expressed and purified by the same method.

### G Protein Activation Assays

A radionucleotide filter binding assay, which measures GDP/guanosine 5′-3-O-(thio)triphosphate (GTPγS) exchange by G protein, was carried out at 20°C as described previously [19,20]. Briefly, purified parapinopsin was mixed with the assay mixture, which consisted of 50 mM HEPES (pH 6.5), 140 mM NaCl, 8 mM MgCl_2_, 1 mM DTT, 1 μM [^35^S]GTPγS, and 4 μM GDP. The mixed samples were irradiated or stored in the dark and immediately mixed with transducin purified from bovine retinas as previously described [19]. UV light (UTVAF-50S-36U-HEAT; Sigma Koki) and/or orange light (O53 cutoff filter; Toshiba) were used for light irradiation. The bovine rhodopsin was irradiated with >520 nm light (Y52 cutoff filter; Toshiba).

### Isolation of cDNAs

Total RNAs from the pineal organs, retinas, and brains of the pufferfish and the retinas of the lamprey were extracted using Sepasol(R)-RNA I (Nacalai Tesque) and were reverse-transcribed to cDNA using an oligo (dT) primer and Superscript III (Invitrogen). The cDNAs were used as a template for PCR amplification. The DNA sequences of seven types of the pufferfish Gi-type G proteins alpha subunits, Gαi-1-1, Gαi-1-2, Gαi-2-1, Gαi-2-2, Gαi-3, Gαo, and Gαz, and rod and cone transducin alpha subunits (Gαt1 and Gαt2) were obtained by analyzing the database of pufferfish. Further, partial cDNAs of these Gα proteins were then isolated from the pufferfish retina, pineal, and brain by PCR amplification with gene specific primers designed using the databases. Partial cDNAs of the transducin alpha subunits (GαtS and GαtL) were isolated from the lamprey retina cDNA by PCR amplification with gene specific primers, designed according to the genome sequences of the sea lamprey *Petromyzon marinus* transducins [18]. The sequences reported in this paper have been deposited in the DDBJ database [accession nos. LC062624 (GαtS), LC062625 (GαtL)].

### Antibodies

Mouse polyclonal antibodies to the pufferfish Gi-type G proteins were generated against the helical domain of nine types of the pufferfish Gαi (119–120 amino acids), Gαt1 (Q54-R172), Gαt2 (Q54-R172), Gαi-1-1 (E58-R176), Gαi-1-2 (E58-R176), Gαi-2-1 (E58-R177), Gαi-2-2 (E58-R177), Gαi-3 (E58-R176), Gαo (E58-R177), and Gαz (S58-R177), using the pMAL protein fusion and purification system (New England Biolabs) [21]. The rabbit anti-Gαq polyclonal antibody was a gift from Dr. Tatsuo Suzuki [22], and the mouse anti-Gαt monoclonal antibody (TF15; CytoSignal) and the rabbit anti-Gαs polyclonal antibody (K-20; Santa Cruz Biotechnology) were commercially obtained. In addition, the rabbit anti-parapinopsin polyclonal antibodies of pufferfish and zebrafish were generated against the C-terminal regions of parapinopsins (PP1) [8]. The rabbit anti-parapinopsin polyclonal antibody of lamprey was used as in our previous report [21].

The specific immunoreactivity of each antibody was examined by immunoblot analysis. The cDNAs encoding the helical domain of the pufferfish Gαt1 and Gαt2 were inserted into the expression vector pQE40 (Qiagen), and the Gαt1 and Gαt2 fragments tagged by 6 × His were expressed in *Escherichia coli* and purified by the ProBond Purification System (Life Technologies). Proteins were separated by SDS-PAGE, transferred onto a PVDF membrane, and incubated with antibodies to the pufferfish Gt1 and Gt2 (diluted 1:500). Immunoreactivity was detected using VECTASTAIN ABC Kit (Vector Laboratories).

### Immunohistochemistry

Eyes and pineal organs, to which a small piece of adjacent tissue was attached, were isolated from the animals. The samples were fixed in 4% paraformaldehyde in 0.1 M sodium phosphate buffer (PB, pH 7.4) at 4°C overnight. Each organ was cryoprotected by immersion in 0.1 M PB containing 15% and 30% sucrose, embedded in OCT compound (Sakura), and sectioned at 20 μm on a cryostat (HM 520; Microm International GmbH).

Immunohistochemical analyses were conducted as previously described [21]. Briefly, tissue sections were incubated with the primary antibodies (diluted 1:500) and subsequently incubated with Alexa Fluor 488- or Alexa Fluor 594-conjugated anti-mouse or anti-rabbit IgG (diluted 1:500; Invitrogen) for immunofluorescent detection. We examined the stained sections under a fluorescence microscope (Leica DM6000 B; Leica Microsystems).

### *In situ* Hybridization

Digoxigenin-labeled antisense RNA probes for the lamprey transducins (GtS and GtL) and lamprey parapinopsin were synthesized using the DIG RNA labeling kit (Roche Applied Science), as previously reported [23]. Briefly, the sections were pretreated with proteinase K and hybridized with the antisense RNA probe diluted in Ultrahyb-Ultrasensitive Hybridization Buffer (Ambion) at 68°C overnight. The probe on the sections was detected using alkaline phosphatase-conjugated anti-digoxigenin (Roche Applied Science), followed by a blue 5-bromo-4-chloro-3-indolyl phosphate nitroblue tetrazolium color reaction.

### GloSensor Assay

The changes in the intracellular cAMP concentration of the pigment-expressing HEK293S cells were measured using the GloSensor cAMP assay (Promega) as previously described [24]. The expression constructs for the parapinopsin were cotransfected with the pGloSensor-22F cAMP plasmid (Promega). The transfected cells were incubated overnight in the culture medium containing 10% fetal bovine serum (FBS) with 11-*cis* retinal. Prior to the measurements, the culture medium was replaced with a CO_2_-independent medium containing 10% FBS and 2% GloSensor cAMP Reagent (Promega). After equilibration with the medium and a steady basal signal was obtained, the cells were treated with 3.5 μM forskolin, a direct activator of adenylyl cyclase, to increase the intracellular cAMP level. Luminescence, representing the amount of cAMP, was measured at 25°C using a GloMax 20/20n Luminometer (Promega). To measure the light-induced change in the cAMP level in the transfected cells, irradiation with UV and green LED light was applied for 5 sec. UV and green LEDs have an emission maximum at approximately 400 nm and 500 nm, respectively. For measuring the dark-incubated samples, the cells were stored in the dark before the measurements.

## Results and Discussion

**Fig 1:**
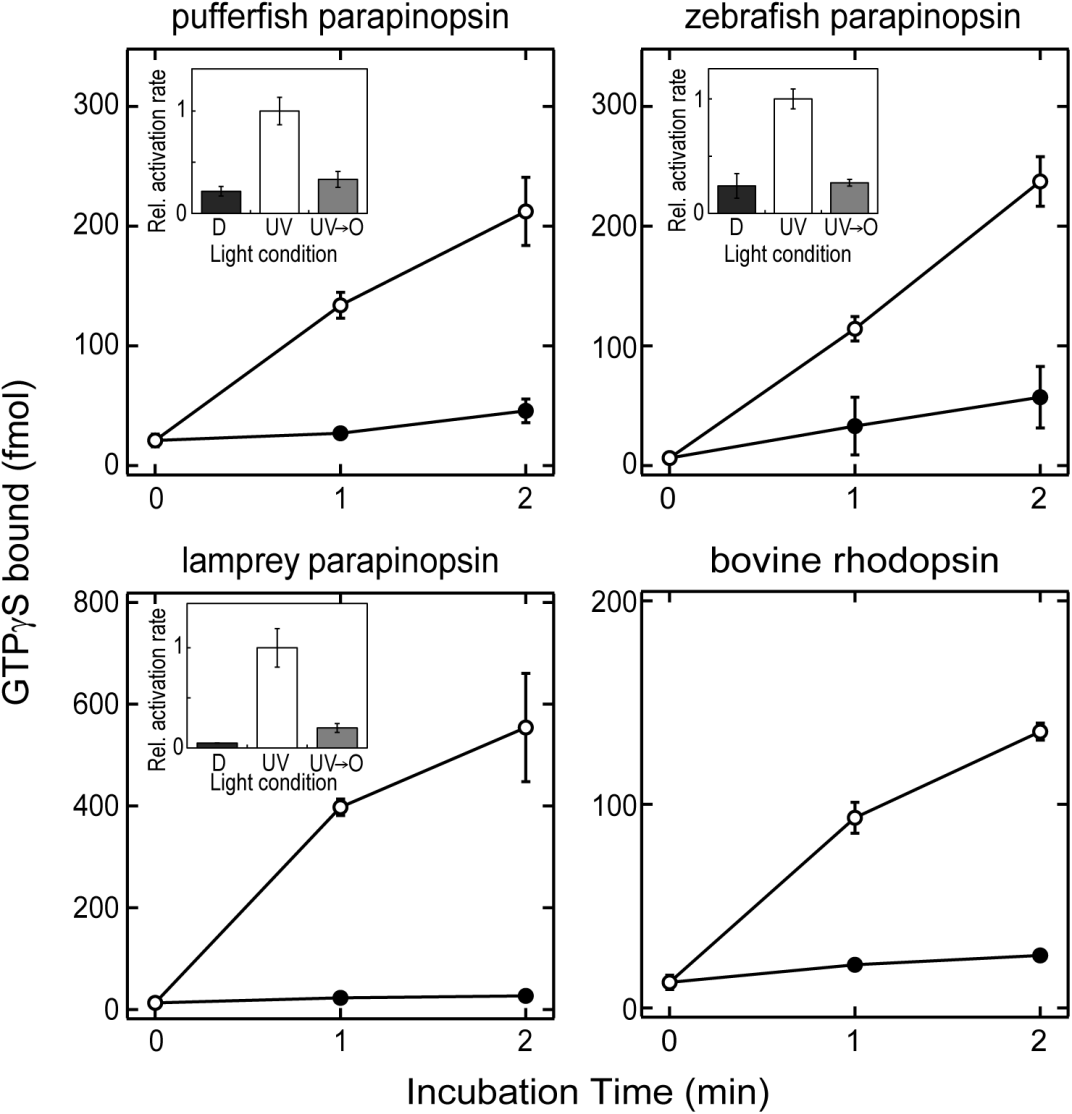
Light-dependent activation of transducin by parapinopsins and bovine rhodopsin *in vitro*. Time courses of the ability of pufferfish, zebrafish, and lamprey parapinopsins and bovine rhodopsin to activate transducin in the dark (filled circles) and after light irradiation (open circles). The transducin activation ability of parapinopsin was measured after irradiation of parapinopsin with ultraviolet (UV) light for 30 s, followed by incubation for 30 s at 20°C for the indicated times. In addition, the bovine rhodopsin that was irradiated with >520 nm light was applied to the same assay. Note that purified parapinopsins (final concentration of 75 nM) or purified bovine rhodopsin (final concentration of 1.5 nM) was used for the assay. (Inset) The transducin activation rates of parapinopsins were compared in the dark (D), after UV-light irradiation (UV) and after additional orange light (>530 nm light) irradiation following UV-light irradiation (UV→O). Data were expressed as means of three separate experiments with standard errors.

We previously reported that parapinopsin was immunohistochemically colocalized with transducin in the lamprey pineal photoreceptor cells [15]. First, we examined whether parapinopsin activated transducin *in vitro* using purified pufferfish, zebrafish, and lamprey parapinopsins. We used purified transducin from bovine retinas, because the amino acid sequence of transducin C-terminal region, which is bound to rhodopsin, is highly conserved among varied vertebrate transducins [25]. Teleost and lamprey parapinopsins activated transducin after the absorption of UV light, whereas parapinopsin stored in the dark (unirradiated parapinopsin) did not exhibit remarkable transducin activation ability (Fig 1), suggesting that parapinopsin activates transducin in a light-dependent manner in the pineal organ. Interestingly, after additional orange light irradiation following UV-light irradiation, parapinopsin did not remarkably activate transducin (Fig 1 inset), which is consistent with the previous spectroscopic observation that the photoproduct of parapinopsin completely reverts to the original dark state by orange light irradiation, showing the bistable nature or the photoregeneration ability [7]. In this experimental condition, a ~50-fold amount of parapinopsins was required to obtain transducin activation efficiency similar to that of bovine rhodopsin. The difference is consistent with our previous report that lamprey parapinopsin as well as bovine rhodopsin can activate Gi-type G protein in *in vitro* experiment, but the activation efficiency of lamprey parapinopsin is lower (1/20–1/50) than that of bovine rhodopsin [13,17].

Our previous immunohistochemical study strongly suggested that parapinopsin was colocalized with transducin in the pineal photoreceptor cells of the lamprey [15]. Various vertebrates contain two types of transducin, Gt1 and Gt2, whereas the lamprey contains the unique transducin GtL, in addition to the Gt1 type transducin GtS [18]. Therefore, we investigated the types of transducins that were co-expressed with parapinopsin in teleost and lamprey pineal organs.

**Fig 2:**
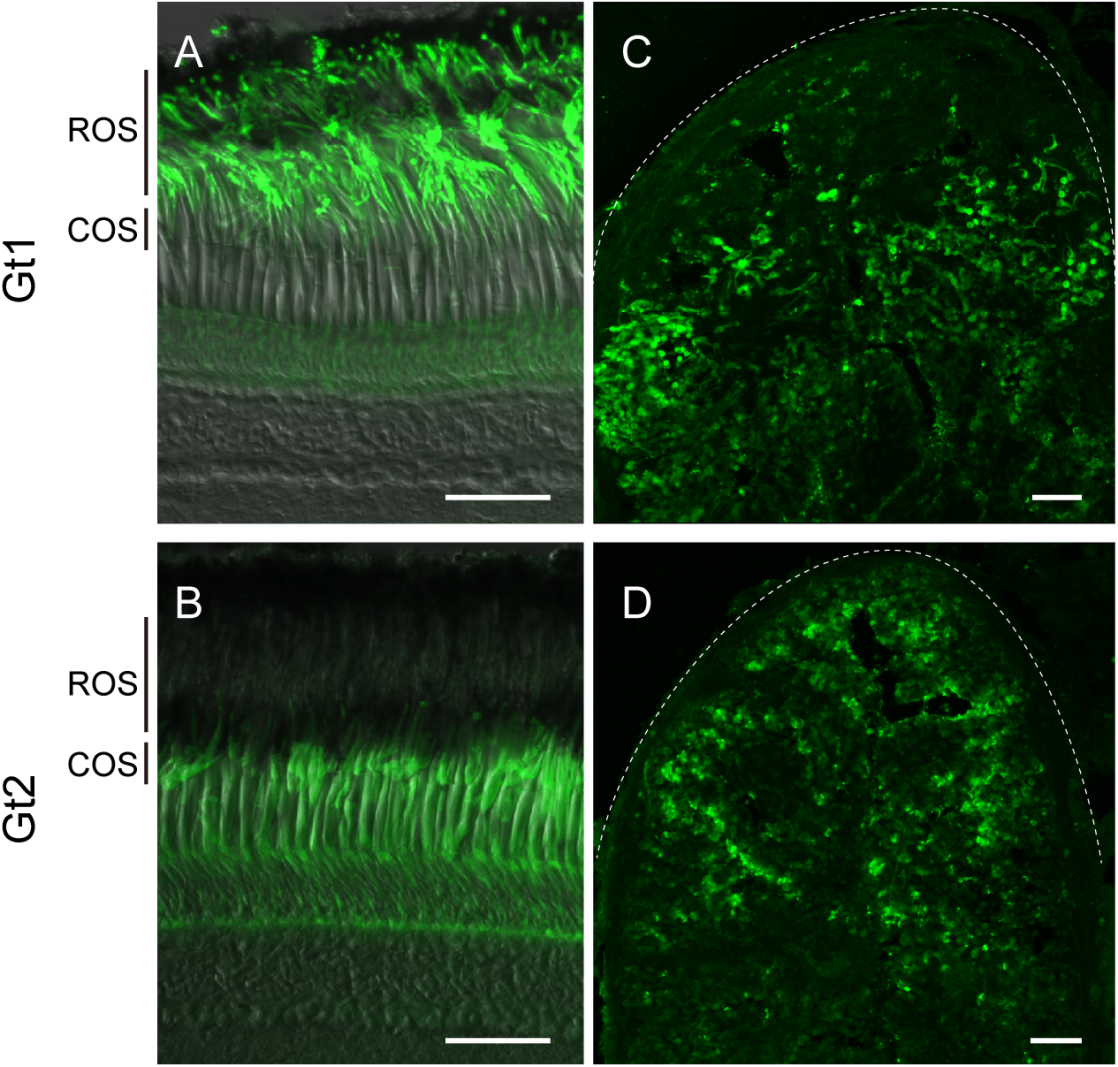
Immunoreactivity of transducins in the pufferfish retina and pineal organ. (A, B) The antibodies against Gt1 (A) and Gt2 (B) specifically stain the outer segments of rod (ROS) and cone (COS) photoreceptor cells, respectively. (C, D) Immunoreactivities of antibody to Gt1 (C) and Gt2 (D) are observed in the pineal organ. Scale bars = 50 μm.

**Fig 3:**
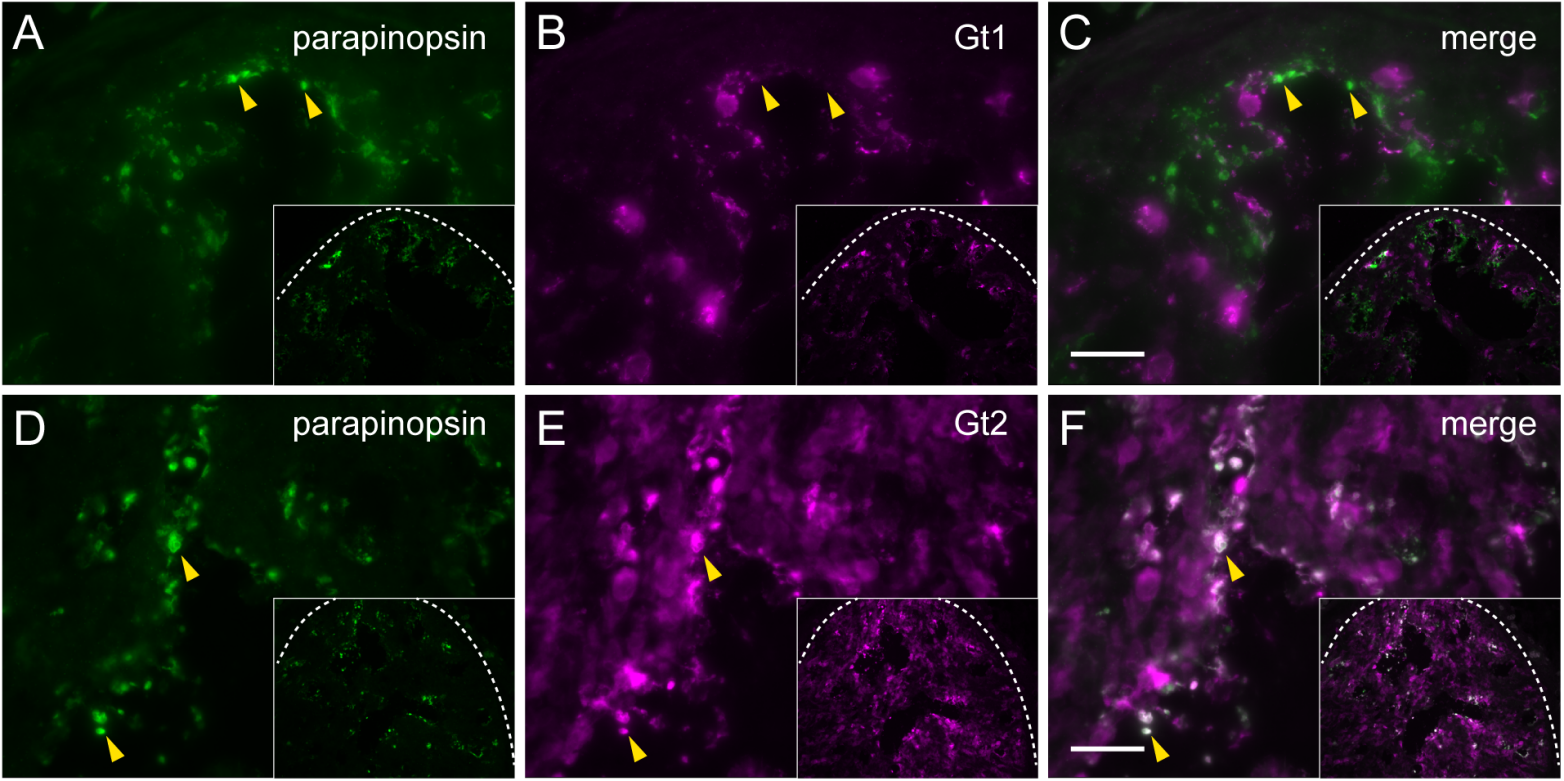
Immunohistochemical localization of parapinopsin and transducins in the pufferfish pineal organ. Parapinopsin (A, green) and Gt1 (B, magenta) are not colocalized (merged image in panel C), whereas parapinopsin (D, green) and Gt2 (E, magenta) are colocalized (merged image in panel F). The arrowheads show the parapinopsin immunoreactivity in the pineal organ. Scale bars = 20 μm. Low magnification images of the pufferfish pineal organ (white dotted traces) are shown in insets.

First, we examined transducin in the pufferfish pineal photoreceptor cells. We generated the specific antibodies to pufferfish Gt1 and Gt2, which specifically stained Gt1 and Gt2 peptides, respectively (S1 Fig), and immunolabeled rod and cone cells in the retina, respectively (Fig 2A and 2B). Immunohistochemical investigation with the antibodies revealed that both Gt1 and Gt2 were distributed in the pineal organs (Fig 2C and 2D). Double immunostaining with antibodies to Gt1 and Gt2 showed that Gt2-immunoreactivity was observed in the parapinopsin containing photoreceptor cells of the pufferfish pineal organ (Fig 3D–3F); however, Gt1-immunoreactivity was not observed (Fig 3A–3C), indicating that parapinopsin is colocalized with Gt2, but not Gt1, in the pineal photoreceptor cells. We further assessed whether the pineal photoreceptor cells contained a high amount of G protein other than transducin. However, we did not detect clear immunoreactivity of Gs, Gq, or Gis (Gi-1-1, Gi-1-2, Gi2-1, Gi2-2, Gi3, Go, or Gz) other than transducin in the pineal organ (S2 Fig). These results suggest that parapinopsin potentially activates Gi [13,17] in addition to Gt (Fig 1) *in vitro* but couples with Gt2-type transducin in the pufferfish pineal organ.

**Fig 4:**
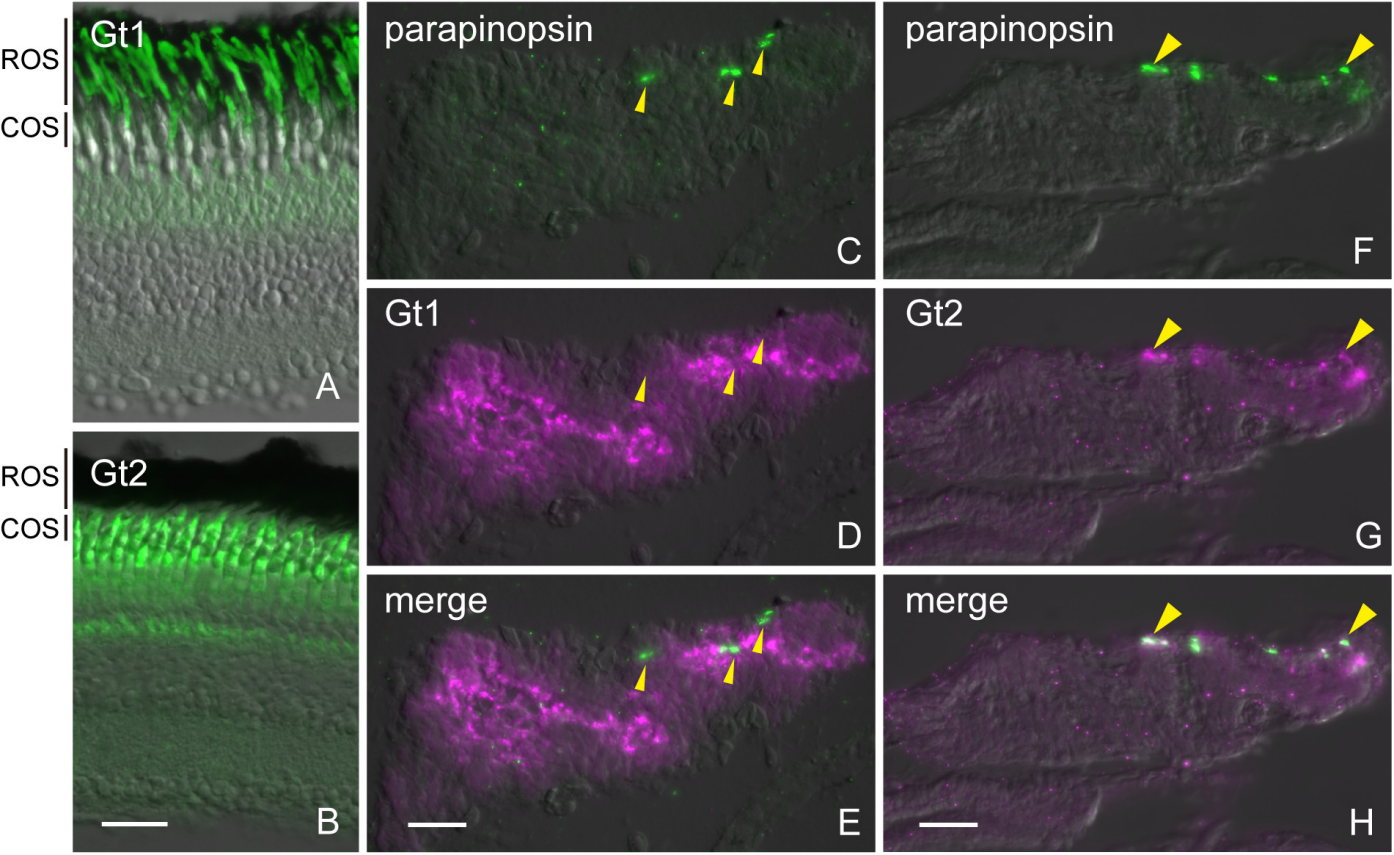
Immunohistochemical localization of transducins in the zebrafish pineal organ. (A, B) The antibodies against Gt1 (A) and Gt2 (B) immunostained the outer segments of rod (ROS) and cone (COS) photoreceptor cells, respectively. (C–E) Parapinopsin (C, green) and Gt1 (D, magenta) are not colocalized (merged image in panel E, arrowhead). (F–H) Parapinopsin (F, green) and Gt2 (G, magenta) are colocalized (merged image in panel H). The arrowheads show the parapinopsin immunoreactivity in the pineal organ. Scale bars = 20 μm.

Furthermore, we investigated whether parapinopsin was colocalized with Gt2 in the zebrafish pineal organ. We confirmed that the antibodies against Gt1 and Gt2 of pufferfish specifically immunostained rod and cone photoreceptor cells in the zebrafish retina, respectively (Fig 4A and 4B). Further, we investigated the distributions of both Gt1 and Gt2 in the zebrafish pineal organ with the antibodies (Fig 4C and 4F). Parapinopsin was colocalized with Gt2 (Fig 4F–4H), but not with Gt1 (Fig 4C–4E), in the zebrafish pineal organ. These results suggest that parapinopsin is coupled to Gt2 in the teleost pineal organ. On the other hand, immunoreactivity to Gt1 was observed in the exorhodopsin-containing photoreceptor cells (S3 Fig).

**Fig 5:**
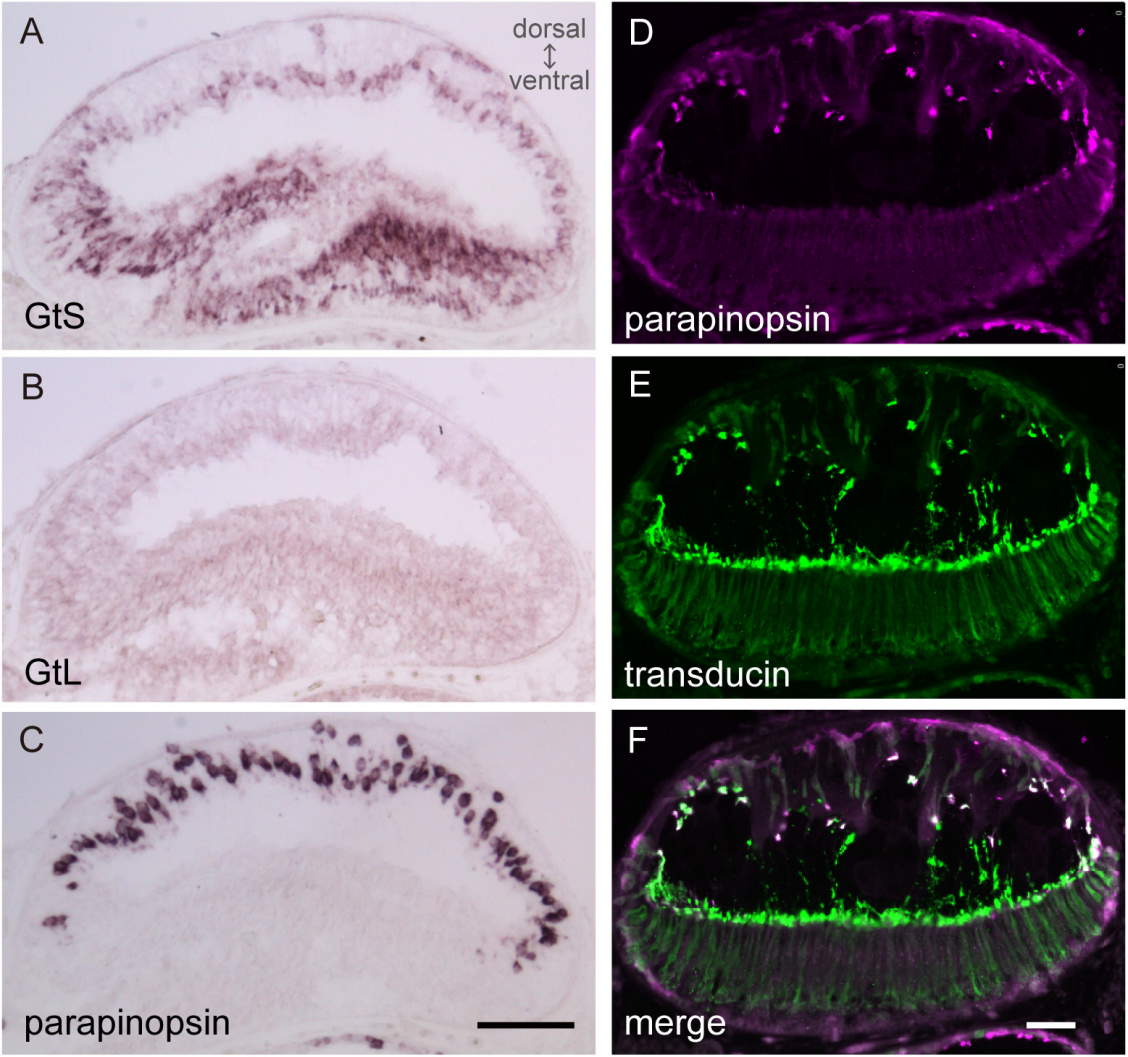
Localization of transducins in the lamprey pineal organ. (A, B) *In situ* hybridizations with the antisense probes of GtS and GtL show that GtS is expressed in the photoreceptor cells of the dorsal and ventral regions (A), but GtL is not detected (B). (C) *In situ* hybridization with the parapinopsin antisense probe shows that parapinopsin is expressed in the photoreceptor cells of the dorsal region. (D) Parapinopsin immunoreactivity (magenta) is localized in the dorsal region of the lamprey pineal organ. (E) Transducin immunoreactivity (green) is distributed in both the dorsal and ventral regions using anti-rod/cone transducin antibody TF15. (F) Parapinopsin and transducin are colocalized in the dorsal photoreceptor cells (white in merged image). Scale bars = 50 μm.

Further, we analyzed the localization of the two types of transducins in the lamprey pineal organ using *in situ* hybridization, which can distinguish between GtS and GtL. GtS was expressed in both dorsal and ventral photoreceptor cells of the lamprey pineal organ (Fig 5A), whereas GtL was not detected (Fig 5B), suggesting that lamprey parapinopsin in the dorsal photoreceptors (Fig 5C) is colocalized with and coupled to GtS (Fig 5).

Our immunohistochemical study indicated that parapinopsin and exorhodopsin, which is close to rhodopsin [26], are colocalized with different types of transducins, Gt2 and Gt1, respectively in the teleost pineal organ (Figs 3 and 4, S3 Fig). The different usage of Gt2 and Gt1 for parapinopsin and exorhodopsin in the teleost pineal organ is similar to that for cone opsins and rhodopsin in the retina, although the physiological implications are unclear. However, in the lamprey pineal organ, *in situ* hybridization suggested that parapinopsin and rhodopsin were coupled to the same transducin GtS in the dorsal and ventral regions (Fig 5), which are involved in wavelength discrimination and sensing light intensity, respectively [21,27]. Muradov et al. (2008) have reported that two types of transducins in the lamprey, GtS and GtL, were expressed in the retinal photoreceptor cells containing rhodopsin and red-sensitive opsin, respectively. GtS is classified into the Gt1 group; however, GtL cannot be clearly classified into either the Gt1 or Gt2 subgroup. The lamprey parapinopsin may employ GtS in place of Gt2 because of a lack of Gt2-type transducin in the lamprey genome. A difference in the signaling property between Gt2 and GtS in the pineal photoreceptors should be investigated in the near future.

Several types of vertebrate non-visual pigments, e. g., pinopsin, VA/VAL opsin, and parietopsin have been investigated for their activating G proteins. Furthermore, it was reported that a pineal pigment, pinopsin, was coupled to transducin [28]. VA/VAL opsin exhibited the significant activation of transducin and Gi-type G protein [29,30], although the type of G proteins that VA/VAL opsin is colocalized with is unclear. It has been reported that parietopsin, first identified in the parietal eye, is colocalized with and coupled to Go-type G protein in the photoreceptor cells of the lizard parietal eye [31]. It was reported that pinopsin, VA/VAL, and parietopsin are “bleaching pigments,” but not bistable pigments, although they are phylogenetically close to parapinopsin [30,32,33]. To the best of our knowledge, this is the first report of a bistable pigment that is colocalized with and activates transducin.

**Fig 6:**
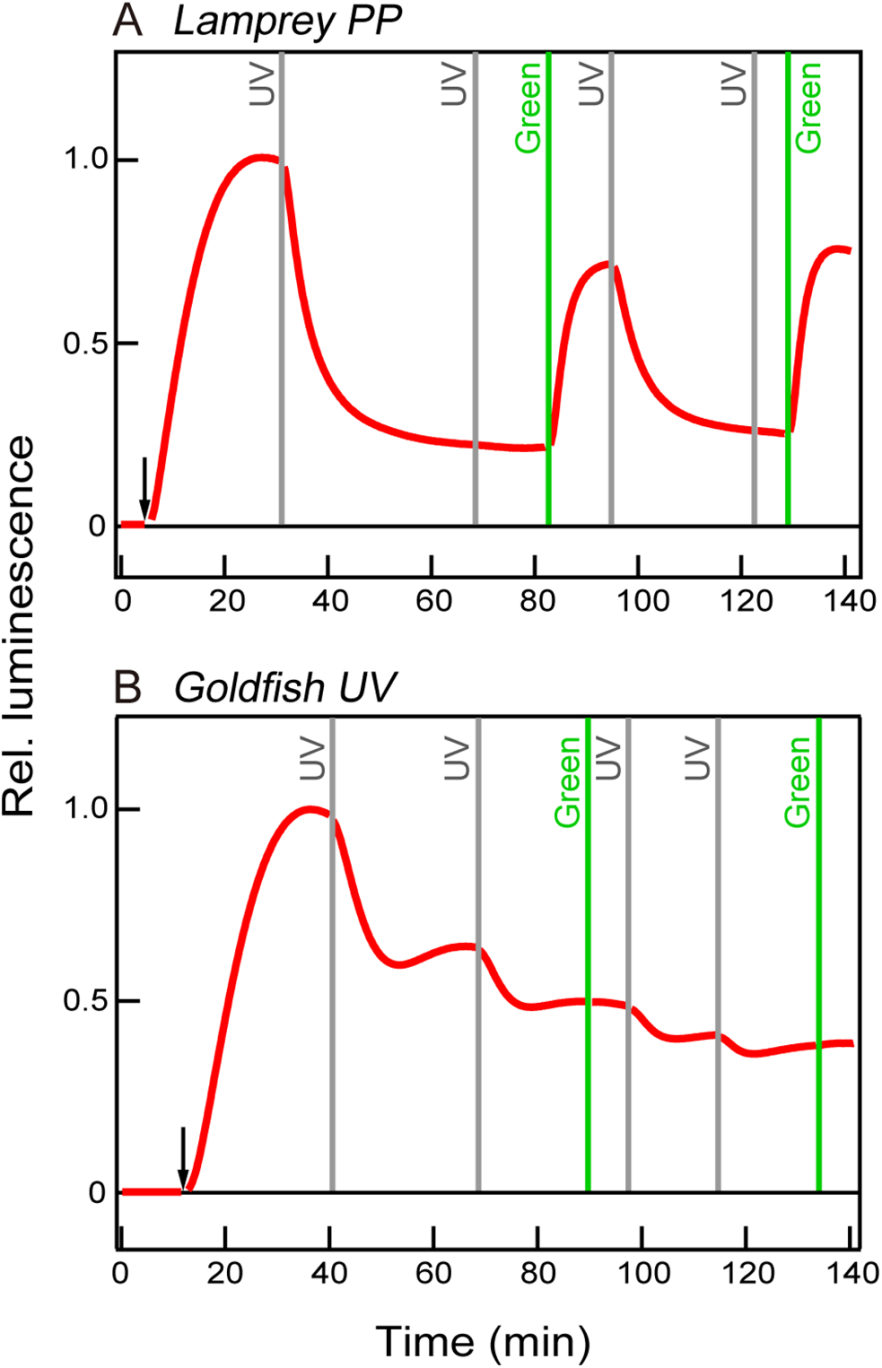
Light-induced cAMP concentration changes in HEK293 cells expressing lamprey parapinopsin or goldfish UV cone visual opsin. Ultraviolet- (UV) light-induced decrease in luminescence signals, which represent the cAMP level, is observed in the lamprey parapinopsin-expressing (A) and the goldfish UV cone visual opsin-expressing (B) HEK293S cells. However, a green light-induced increase (recovery) of cAMP level is found in the lamprey parapinopsin-expressing (A) but not in the goldfish UV cone visual opsin-expressing (B) HEK293S cells. The arrows and vertical lines indicate forskolin treatments and UV and green light irradiations, respectively.

Recently, transducin-coupled opsins, such as bovine rhodopsin, human cone visual pigments, and their mutant opsins, have been used for light-dependent manipulation of targeted cells as an optogenetic tool [34–36]. Our *in vitro* experiment showed that UV and orange lights activate and deactivate parapinopsin, respectively (Fig 1), suggesting that different colors of light may control the cell response using parapinopsin as an optogenetic tool. Therefore, we further investigated the effect of the bistable nature of parapinopsin on the second messenger, cAMP change in the living cells, and compared its change with that in the case of UV-sensitive vertebrate visual pigment, which shares common molecular properties, UV-sensitivity, and transducin/Gi activation ability *in vitro* [13,19]. Interestingly, UV-light irradiation caused a decrease of cAMP level, which was pre-increased by the addition of forskolin, and subsequent green light irradiation resulted in recovery (increase) of cAMP level in the cultured cells expressing parapinopsin (Fig 6A). UV and green light irradiations repeatedly down- and up-regulated intracellular cAMP level, reflecting activation and deactivation of parapinopsin by UV and green light irradiations, respectively [7]. However, rapid recovery of the cAMP level by green light irradiation could not be observed in the cells expressing UV-sensitive goldfish visual pigment (Fig 6B). These findings indicate a possibility that the bistable nature of parapinopsin could contribute to wavelength-dependent manipulation of cell activities as a unique optogenetic tool [37].

It is of interest to discuss acquisition of transducin-coupled visual and non-visual opsins during the course of their evolution. It was suggested that vertebrate visual pigments characterized by a bleaching nature and transducin-coupling ability had evolved from an ancestral pigment characterized by a bistable nature [13]. We have previously reported that Opn3 homologues, which are clearly distinguished from, but phylogenetically close to, both vertebrate non-visual and visual pigments, are the bistable pigments and activate Gi-type and Go-type G proteins but not transducin *in vitro* [24]. Our finding that a bistable non-visual pigment parapinopsin is coupled to transducin suggests that an ancestral bistable pigment similar to parapinopsin had acquired an ability to be coupled to transducin, and then acquired a bleaching property during evolution. We have recently revealed that the lamprey parapinopsin binds to β-arrestin in a light-dependent manner in the pineal photoreceptor cells to completely shut off the stable photoproduct of parapinopsin, involving internalization of the photoproduct. It can be speculated that parapinopsin is deactivated by β-arrestin because of its bistable nature, unlike the combination of visual arrestin and visual pigment having a bleaching property [15]. As described above, in a biochemical assay, a higher amount of pigments was required for parapinopsin than for bovine rhodopsin to activate transducin, showing that transducin activation ability is greater for bovine rhodopsin than for parapinopsin (Fig 1). This observation can be explained by our previous idea that the bleaching property, which involves a greater conformational change of opsin upon light absorption, contributed to an efficient activation of G protein for higher sensitivity of vision [13,20]. It is hypothesized that an ancestor of visual pigments may have acquired the transducin-mediated signal transduction cascade for hyperpolarization, and subsequently, the effective G protein activation ability with a bleaching nature for higher visual sensitivity.

## Supporting Information

S1 Fig Immunoblot analyses showing the specificity of antibodies against Gt1 and Gt2. Gt1 (lanes 2, 4, and 6) or Gt2 (lanes 3, 5, and 7) peptide-containing *Escherichia coli* proteins were applied to immunoblotting using antibodies against Gt1 and Gt2. Lanes 2 and 3 were stained with Coomassie brilliant blue. Lanes 4 and 5 and lanes 6 and 7 were stained with antibodies to Gt1 and Gt2, respectively. The results demonstrate that the antibodies specifically bind Gt1 and Gt2. M indicates molecular weight standard markers (lane 1) (Bio-Rad Laboratories).
(TIF)

S2 Fig Localization of G proteins in the pufferfish pineal organ. (TIF)

S3 Fig Immunohistochemical localization of exorhodopsin and rod transducin in the zebrafish pineal organ. (TIF)

## References

[1] Dodt E. The parietal eye (pineal and parietal organs) of lower vertebrates In: Jung R, editor. Handbook of sensory physiology. Berlin: Springer; 1973 pp. 113–140.

[2] Dodt E, Heerd E. Mode of action of pineal nerve fibers in frogs. J Neurophysiol. 1962; 25: 405–429. 13886872 10.1152/jn.1962.25.3.405

[3] Morita Y. Lead pattern of the pineal neuron of the rainbow trout (*Salmo irideus*) by illumination of the diencephalon. Pflüers Arch. 1966; 289: 155–167. 5237284

[4] Morita Y, Dodt E. Slow photic responses of the isolated pineal organ of lamprey. Nova Acta Leopoldina. 1973; 38: 331–339.

[5] Solessio E, Engbretson GA. Antagonistic Chromatic Mechanisms in Photoreceptors of the Parietal Eye of Lizards. Nature. 1993; 364: 442–445. 8332214 10.1038/364442a0

[6] Blackshaw S, Snyder SH. Parapinopsin, a novel catfish opsin localized to the parapineal organ, defines a new gene family. J Neurosci. 1997; 17: 8083–8092. 9334384 10.1523/JNEUROSCI.17-21-08083.1997 PMC6573767

[7] Koyanagi M, Kawano E, Kinugawa Y, Oishi T, Shichida Y, Tamotsu S, et al Bistable UV pigment in the lamprey pineal. Proc Natl Acad Sci USA. 2004; 101: 6687–6691. 15096614 10.1073/pnas.0400819101 PMC404106

[8] Koyanagi M, Wada S, Kawano-Yamashita E, Hara Y, Kuraku S, Kosaka S, et al Diversification of non-visual photopigment parapinopsin in spectral sensitivity for diverse pineal functions. BMC Biol. 2015; 13: 73 10.1186/s12915-015-0174-9 26370232 PMC4570685

[9] Wada S, Kawano-Yamashita E, Koyanagi M, Terakita A. Expression of UV-sensitive parapinopsin in the iguana parietal eyes and its implication in UV-sensitivity in vertebrate pineal-related organs. PLOS ONE. 2012; 7: e39003 10.1371/journal.pone.0039003 22720013 PMC3375259

[10] Terakita A. The opsins. Genome Biol. 2005; 6: 213 15774036 10.1186/gb-2005-6-3-213 PMC1088937

[11] Koyanagi M, Kubokawa K, Tsukamoto H, Shichida Y, Terakita A. Cephalochordate melanopsin: evolutionary linkage between invertebrate visual cells and vertebrate photosensitive retinal ganglion cells. Curr Biol. 2005; 15: 1065–1069. 15936279 10.1016/j.cub.2005.04.063

[12] Koyanagi M, Terakita A. Gq-coupled rhodopsin subfamily composed of invertebrate visual pigment and melanopsin. Photochem Photobiol. 2008; 84: 1024–1030. 10.1111/j.1751-1097.2008.00369.x 18513236

[13] Terakita A, Koyanagi M, Tsukamoto H, Yamashita T, Miyata T, Shichida Y. Counterion displacement in the molecular evolution of the rhodopsin family. Nat Struct Mol Biol. 2004; 11: 284–289. 14981504 10.1038/nsmb731

[14] Tsukamoto H, Terakita A. Diversity and functional properties of bistable pigments. Photochem Photobiol Sci. 2010; 9: 1435–1443. 10.1039/c0pp00168f 20852774

[15] Kawano-Yamashita E, Koyanagi M, Shichida Y, Oishi T, Tamotsu S, Terakita A. Beta-arrestin functionally regulates the non-bleaching pigment parapinopsin in lamprey pineal. PLOS ONE. 2011; 6: e16402 10.1371/journal.pone.0016402 21305016 PMC3031554

[16] Lohse MJ, Benovic JL, Codina J, Caron MG, Lefkowitz RJ. Beta-Arrestin: a protein that regulates beta-adrenergic receptor function. Science. 1990; 248: 1547–1550. 2163110 10.1126/science.2163110

[17] Tsukamoto H, Terakita A, Shichida Y. A pivot between helices V and VI near the retinal-binding site is necessary for activation in rhodopsins. J Biol Chem. 2010; 285: 7351–7357. 10.1074/jbc.M109.078709 20053991 PMC2844183

[18] Muradov H, Kerov V, Boyd KK, Artemyev NO. Unique transducins expressed in long and short photoreceptors of lamprey *Petromyzon marinus* . Vision Res. 2008; 48: 2302–2308. 10.1016/j.visres.2008.07.006 18687354 PMC2613798

[19] Terakita A, Yamashita T, Nimbari N, Kojima D, Shichida Y. Functional interaction between bovine rhodopsin and G protein transducin. J Biol Chem. 2002; 277: 40–46. 11606568 10.1074/jbc.M104960200

[20] Tsukamoto H, Farrens DL, Koyanagi M, Terakita A. The magnitude of the light-induced conformational change in different rhodopsins correlates with their ability to activate G proteins. J Biol Chem. 2009; 284: 20676–20683. 10.1074/jbc.M109.016212 19497849 PMC2742832

[21] Kawano-Yamashita E, Terakita A, Koyanagi M, Shichida Y, Oishi T, Tamotsu S. Immunohistochemical characterization of a parapinopsin-containing photoreceptor cell involved in the ultraviolet/green discrimination in the pineal organ of the river lamprey *Lethenteron japonicum* . J Exp Biol. 2007; 210: 3821–3829. 17951423 10.1242/jeb.007161

[22] Nagata T, Koyanagi M, Tsukamoto H, Saeki S, Isono K, Shichida Y, et al Depth perception from image defocus in a jumping spider. Science. 2012; 335: 469–471. 10.1126/science.1211667 22282813

[23] Sun L, Kawano-Yamashita E, Nagata T, Tsukamoto H, Furutani Y, Koyanagi M, et al Distribution of mammalian-like melanopsin in cyclostome retinas exhibiting a different extent of visual functions. PLOS ONE. 2014; 9: e108209 10.1371/journal.pone.0108209 25251771 PMC4177573

[24] Koyanagi M, Takada E, Nagata T, Tsukamoto H, Terakita A. Homologs of vertebrate Opn3 potentially serve as a light sensor in nonphotoreceptive tissue. Proc Natl Acad Sci U S A. 2013; 110: 4998–5003. 10.1073/pnas.1219416110 23479626 PMC3612648

[25] Simon MI, Strathmann MP, Gautam N. Diversity of G proteins in signal transduction. Science. 1991; 252: 802–808. 1902986 10.1126/science.1902986

[26] Mano H, Kojima D, Fukada Y. Exo-rhodopsin: a novel rhodopsin expressed in the zebrafish pineal gland. Brain Res Mol Brain Res. 1999; 73: 110–118. 10581404 10.1016/s0169-328x(99)00242-9

[27] Kawano-Yamashita E, Koyanagi M, Terakita A. The evolution and diversity of pineal and parapineal photopigments In: Hunt DM, Hankins MW, Collin SP, Marshall NJ, editors. Evolution of visual and non-visual pigments: Springer; 2014 pp. 1–21.

[28] Kasahara T, Okano T, Yoshikawa T, Yamazaki K, Fukada Y. Rod-type transducin alpha-subunit mediates a phototransduction pathway in the chicken pineal gland. J Neurochem. 2000; 75: 217–224. 10854264 10.1046/j.1471-4159.2000.0750217.x

[29] Friedmann D, Hoagland A, Berlin S, Isacoff EY. A spinal opsin controls early neural activity and drives a behavioral light response. Curr Biol. 2015; 25: 69–74. 10.1016/j.cub.2014.10.055 25484291 PMC4286461

[30] Sato K, Yamashita T, Ohuchi H, Shichida Y. Vertebrate ancient-long opsin has molecular properties intermediate between those of vertebrate and invertebrate visual pigments. Biochemistry. 2011; 50: 10484–10490. 10.1021/bi201212z 22066464

[31] Su CY, Luo DG, Terakita A, Shichida Y, Liao HW, Kazmi MA, et al Parietal-eye phototransduction components and their potential evolutionary implications. Science. 2006; 311: 1617–1621. 16543463 10.1126/science.1123802

[32] Nakamura A, Kojima D, Okano T, Imai H, Terakita A, Shichida Y, et al Regulatory mechanism for the stability of the meta II intermediate of pinopsin. J Biochem. 2001; 129: 329–334. 11173536 10.1093/oxfordjournals.jbchem.a002861

[33] Sakai K, Imamoto Y, Su CY, Tsukamoto H, Yamashita T, Terakita A, et al Photochemical nature of parietopsin. Biochemistry. 2012; 51: 1933–1941. 10.1021/bi2018283 22303823 PMC3315353

[34] Airan RD, Thompson KR, Fenno LE, Bernstein H, Deisseroth K. Temporally precise in vivo control of intracellular signalling. Nature. 2009; 458: 1025–1029. 10.1038/nature07926 19295515

[35] Gutierrez DV, Mark MD, Masseck O, Maejima T, Kuckelsberg D, Hyde RA, et al Optogenetic control of motor coordination by Gi/o protein-coupled vertebrate rhodopsin in cerebellar Purkinje cells. J Biol Chem. 2011; 286: 25848–25858. 10.1074/jbc.M111.253674 21628464 PMC3138296

[36] Masseck OA, Spoida K, Dalkara D, Maejima T, Rubelowski JM, Wallhorn L, et al Vertebrate cone opsins enable sustained and highly sensitive rapid control of Gi/o signaling in anxiety circuitry. Neuron. 2014; 81: 1263–1273. 10.1016/j.neuron.2014.01.041 24656249

[37] Koyanagi M, Terakita A. Diversity of animal opsin-based pigments and their optogenetic potential. Biochim Biophys Acta. 2014; 1837: 710–716. 10.1016/j.bbabio.2013.09.003 24041647

