# Supplementary figures and images for: Activation of Transducin by Bistable Pigment Parapinopsin in the Pineal Organ of Lower Vertebrates
Source: PLoS One. 2015 Oct 22;10(10):e0141280. doi: 10.1371/journal.pone.0141280 (PMC4619617; doi:10.1371/journal.pone.0141280)

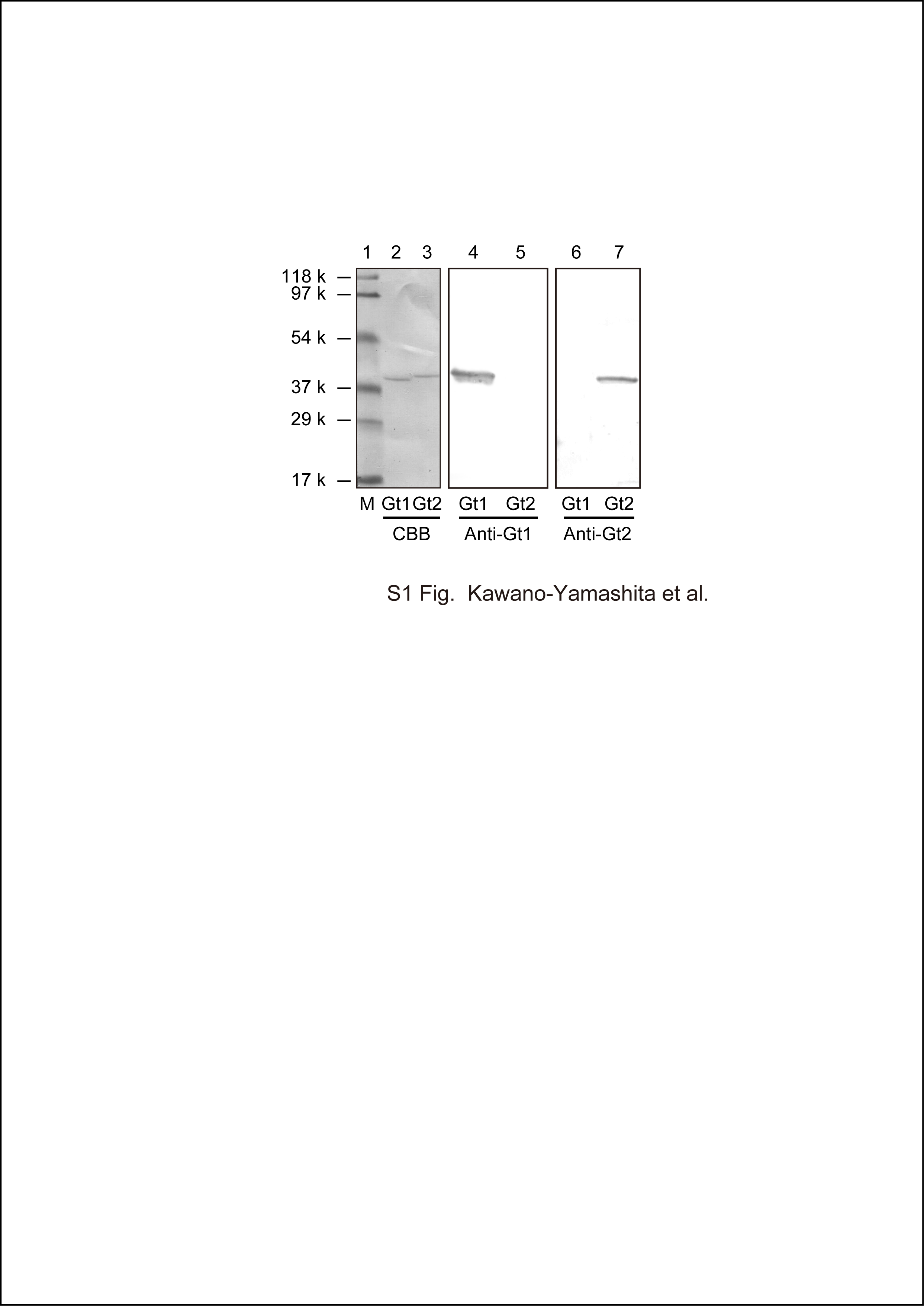

Supplement: S1 Fig — Gt1 (lanes 2, 4, and 6) or Gt2 (lanes 3, 5, and 7) peptide-containing Escherichia coli proteins were applied to immunoblotting using antibodies against Gt1 and Gt2. Lanes 2 and 3 were stained with Coomassie brilliant blue. Lanes 4 and 5 and lanes 6 and 7 were stained with antibodies to Gt1 and Gt2, respectively. The results demonstrate that the antibodies specifically bind Gt1 and Gt2. M indicates molecular weight standard markers (lane 1) (Bio-Rad Laboratories). (TIF) [file pone.0141280.s001.tif]

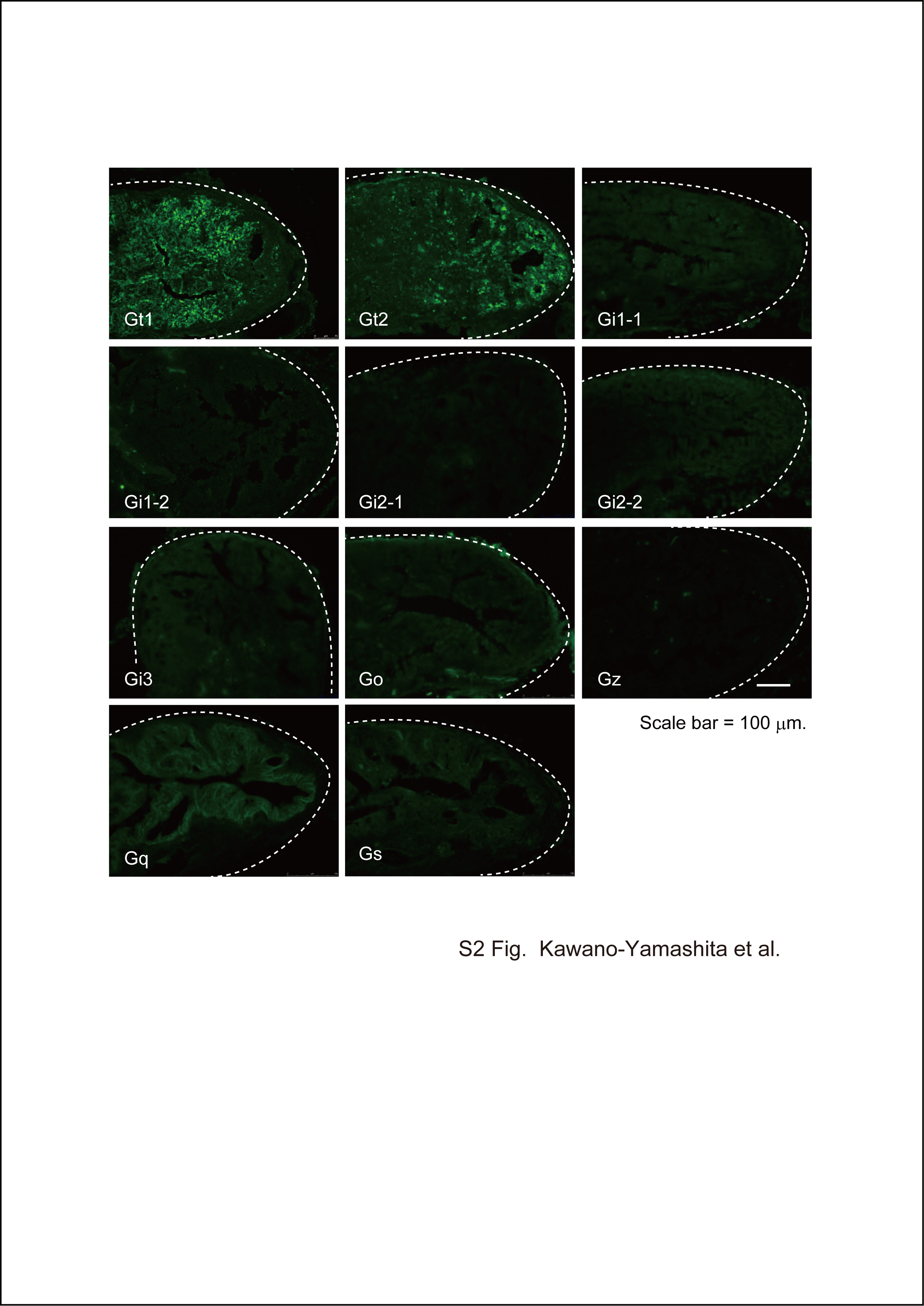

Supplement: S2 Fig — (TIF) [file pone.0141280.s002.tif]

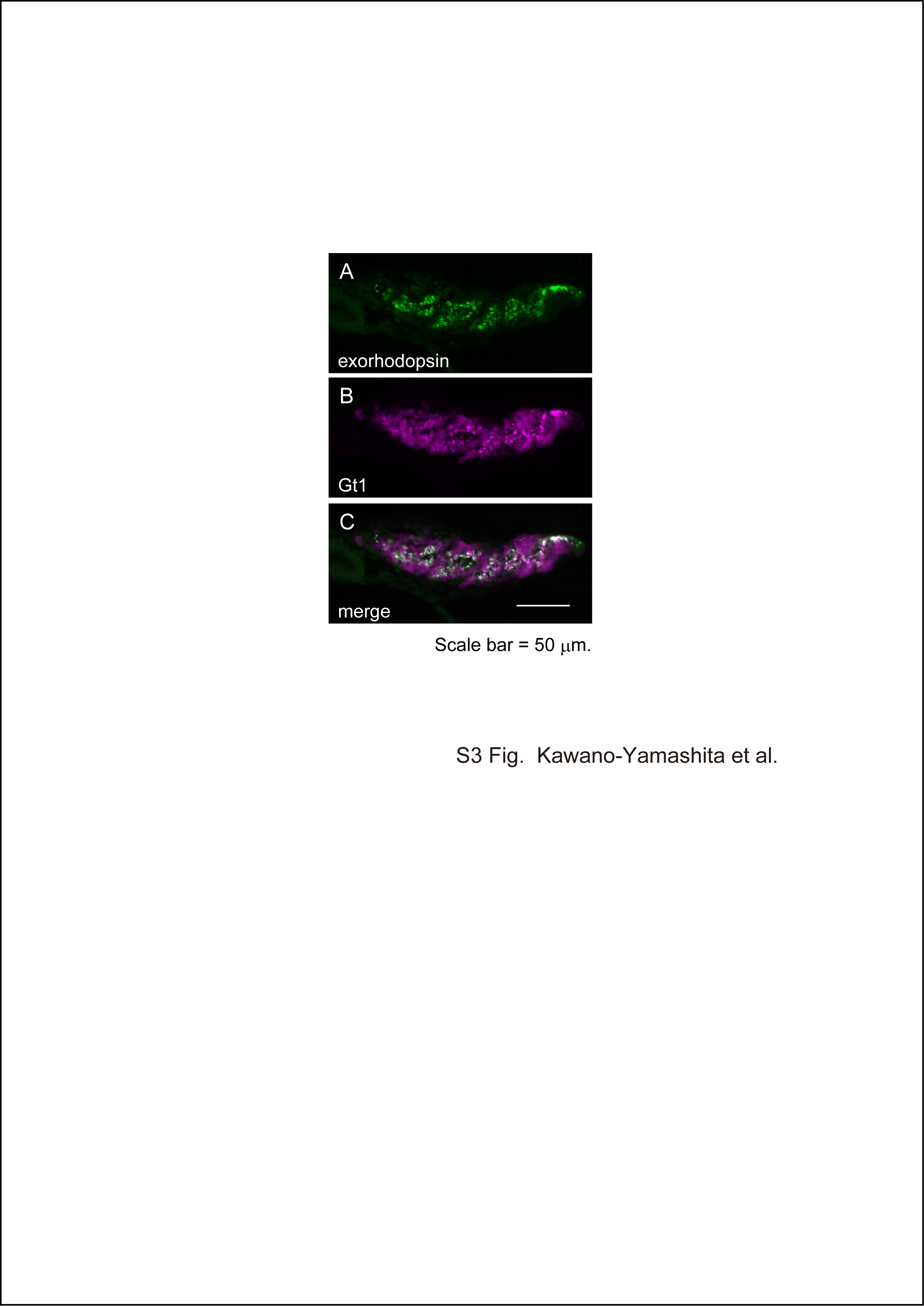

Supplement: S3 Fig — (TIF) [file pone.0141280.s003.tif]
